# Chiari malformation type I with cervicothoracic syringomyelia masquerading as bibrachial amyotrophy: a case report

**DOI:** 10.1186/1752-1947-9-11

**Published:** 2015-01-27

**Authors:** Jeffrey R Mora, Richard A Rison, Said R Beydoun

**Affiliations:** 5 Keck School of Medicine, Neuromuscular Division, University of Southern California, 1520 San Pablo Street, Suite 3000, Los Angeles, CA 90033 USA; 6 Department of Neurology, University of Southern California Keck School of Medicine, Los Angeles County Medical Center, 1520 San Pablo Street, Suite 3000, Los Angeles, CA 90033 USA; 7 Department of Neurology, PIH Health Hospital-Whittier, 12401 Washington Boulevard, Whittier, California 90602 USA; 8 Keck School of Medicine, Los Angeles County Medical Center, University of Southern California, 1520 San Pablo Street, Suite 3000, Los Angeles, CA 90033 USA

**Keywords:** Atrophy, Bibrachial amyotrophy, Bulbar dysfunction, Cervicothoracic, Chiari type 1 malformation, Distal muscle, Misdiagnosis, Syringomyelia

## Abstract

**Introduction:**

Clinical presentation of syringomyelia can mimic a variety of neuromuscular disorders. A misdiagnosis can result in progressive pressure on the spinal cord, causing the development of severe irreversible neurologic deficits.

**Case presentation:**

We report the very unusual case of a 50-year-old Latino man who developed severe distal muscle atrophy and bulbar dysfunction as a result of Chiari malformation type I with chronic cervicothoracic syringomyelia.

**Conclusion:**

Syringomyelia is a potentially serious neurologic condition with symptoms that can mimic other neuromuscular disorders. Severe untreated cases can result in irreversible spinal cord injury. Prompt diagnosis with magnetic resonance imaging is important in both establishing diagnosis and directing further surgical management.

## Introduction

Syringomyelia results from the blockage of normal cerebrospinal fluid flow through the spinal cord due to either a congenital anatomic anomaly or an acquired structural abnormality. Depending on the severity, location and extent of the underlying syrinx, it can range clinically from an asymptomatic radiologic finding to a neurologically debilitating condition with irreversible spinal cord injury. The presenting symptoms and clinical course may vary; as a result, syringomyelia may mimic other neuromuscular disorders and be misdiagnosed.

## Case presentation

### Patient history

A 50-year-old right-handed Latino man presented with worsening of previously stable chronic motor symptoms. He had initially developed sudden weakness and difficulty using his right upper extremity at the age of 16 years. He eventually had to learn to write using his left hand because of the severity of his weakness. His symptoms progressed over several years, eventually developing into severe symmetrical upper extremity weakness and atrophy in his distal muscles. His lower extremities were spared. Our patient stated he was diagnosed with a type of muscular dystrophy in the US when he was 25 years old. After receiving this diagnosis, he subsequently had a muscle biopsy in Mexico that reportedly confirmed his diagnosis. He had no family members with muscular dystrophy or any other neuromuscular conditions. His symptoms reached their plateau at this time. Because of the stability of his symptoms and his understanding of the disease course, our patient did not seek any further medical care for over 20 years. Upon returning to the US, he was seen by a primary care physician and referred for neurological examination to establish care as well as evaluate the worsening of previously stable chronic symptoms.

On presentation, our patient reported a few months of clinical deterioration, particularly recent left upper arm intermittent mild pressure pain. Our patient grew concerned when he noticed increased difficulty performing fine motor tasks with his left hand. In addition, he was experiencing recurrent intermittent episodes of dysphagia while eating solid foods. Our patient denied ever having any previous bulbar symptoms prior to his recent clinical deterioration.

### Physical examination

**Figure 1:**
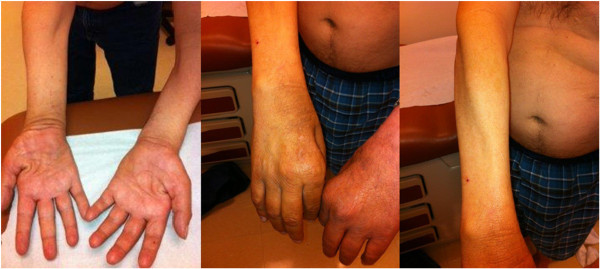
**Physical examination.** Upper extremities with severe asymmetric atrophy of forearm extensors and flexors, with sparing of the brachioradialis muscles.

A clinical examination demonstrated that our patient was thin with significant bilateral upper extremity muscle atrophy and associated weakness. He had severe asymmetric focal segmental atrophy of his bilateral forearm flexor and extensor muscle groups, with preserved and prominent bilateral brachioradialis muscles (Figure 1). He had corresponding severe weakness in his upper extremities as measured using the Medical Research Council Scale, with 4-/5 to 5/5 proximal strength and as low as 1/5 strength in his distal muscles. He had bilateral radial deviation during wrist extension. Although his lower extremities demonstrated normal bulk, he had mild weakness on manual muscle testing as well. Our patient had 4/5 strength in his left hip extensors and flexors as well as 4/5 strength in bilateral knee extensors. He had no face weakness, no sensory deficits, no abnormal reflexes, no muscle fasciculations, and no upper motor neuron signs on examination.

### Electrophysiology

Compound muscle action potentials (CMAP) of his left median, ulnar and radial nerves were absent. His upper extremity sensory nerve action potential (SNAP) responses were normal except for a slightly low amplitude of his ulnar and radial nerves. His lower extremity peroneal and tibial CMAPs and sural SNAPs were normal. Electromyography (EMG) of his upper extremities demonstrated evidence of diffuse chronic neurogenic changes in C5 to T1 innervated muscles as well as evidence of active denervation in his right triceps brachii. In his left lower extremity, chronic neurogenic changes were noted in the gastrocnemius medial head. His left cervical paraspinal muscles were normal on EMG.

### Neuroimaging

**Figure 2:**
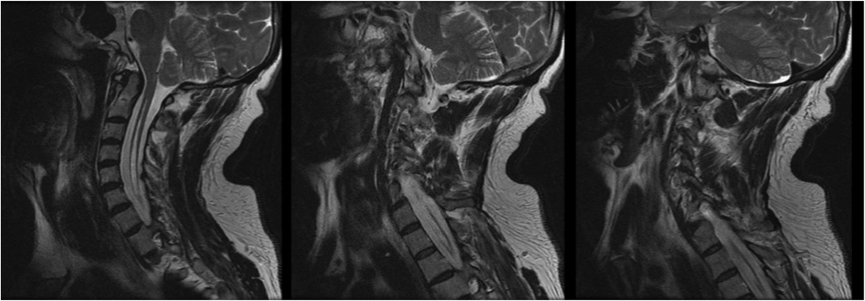
**Magnetic resonance imaging of the cervical spine.** Sequential T2 fast recovery fast spin echo sequence sagittal cuts demonstrating the syrinx from the level of C1 extending inferiorly into the thoracic spinal cord.

**Figure 3:**
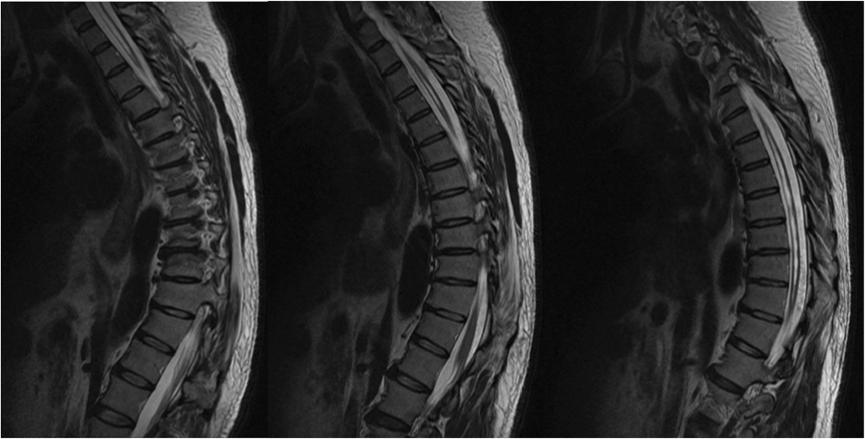
**Magnetic resonance imaging of the thoracic spine.** Sequential T2 fast recovery fast spin echo sequence sagittal cuts demonstrating the syrinx extending inferiorly down to T11 level.

**Figure 4:**
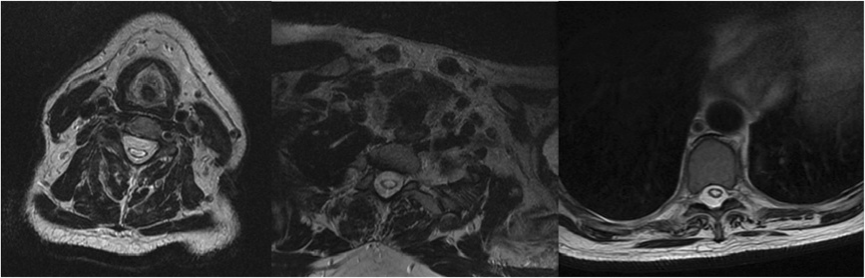
**Magnetic resonance imaging of the cervicothoracic spine.** Axial T2 fast recovery fast spin echo; the syrinx is visible within the central canal of the spinal cord.

**Figure 5:**
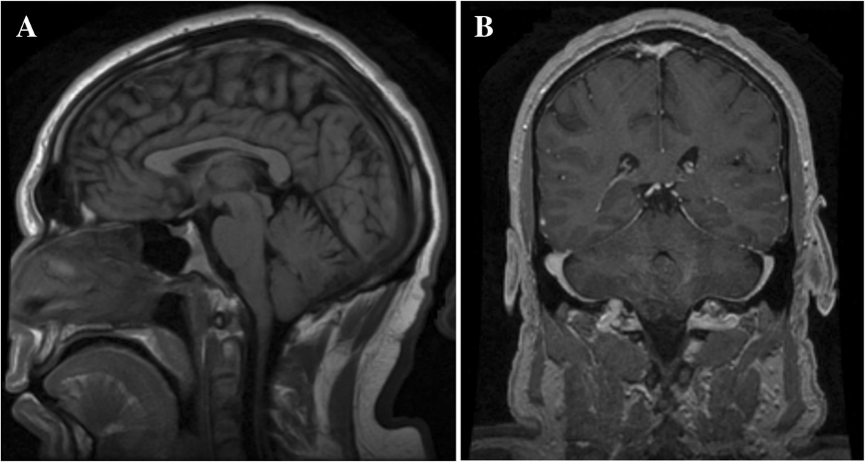
**Magnetic resonance imaging of the brain.** Chiari malformation type I. **(A)** Sagittal T2 fluid-attenuated inversion recovery sequence. **(B)** Coronal spoiled gradient recalled echo (SPGR) post-contrast sequence.

Magnetic resonance imaging (MRI) without contrast of his cervical, thoracic and lumbar spinal cord demonstrated Chiari malformation type I with associated syringohydromyelia extending from C1 (Figure 2) to T11 (Figure 3). The maximal anterior-posterior diameter was 4mm and maximal lateral diameter 10mm. There was cord atrophy and mild atrophy of his paraspinal musculature (Figure 4). Once the syrinx was identified on MRI, our patient was referred to our neurosurgery team for evaluation. An MRI of his brain with and without contrast provided additional views of the Chiari malformation type I (Figure 5).

## Discussion

Syringomyelia is a condition where a cyst, usually composed of excess cerebrospinal fluid (CSF), forms within the spinal cord. Although syrinxes commonly form as a focal dilation of the central canal, they can also form within the spinal cord parenchyma [1]. These lesions can develop in association with a variety of both congenital anatomic anomalies and acquired structural abnormalities, including scoliosis, spina bifida, Chiari malformations, tumors and hemorrhage, as well as post-infectious, post-inflammatory and post-traumatic conditions [2]. Syringomyelia can be further classified based on the pathological and magnetic resonance characteristics of various spinal cord cysts and divided into five groups: non-communicating, communicating, primary parenchymal cavitations, atrophic cavitations and neoplastic cavitations [3].

Chiari malformation type I, a type of non-communicating syringomyelia, is the most common cause of this condition [3]. Imaging of patients with this disorder reveals cerebellar tonsilar ectopia that may protrude through the foramen magnum. The manner in which this simple anatomic anomaly results in cavitation within the spinal cord remains poorly understood, despite the multiple theories that have been proposed following radiographic and pathologic analyses, flow dynamics studies, and experimental animal models. Possible theories for the pathogenesis of syringomyelia secondary to Chiari malformation type I include CSF flow from the fourth canal due to arterial pulsation, with the central canal acting as a one-way valve, or a relative pressure dissociation between the intracranial and spinal subarachnoid spaces; CSF flow from the spinal subarachnoid space via perivascular spaces; and extracellular fluid from spinal cord microcirculation due to disturbed extracellular fluid absorption through intramedullary venous channels [4]. Because of the sensitivity to pressure, symptoms may worsen with the Valsalva maneuver or other activities that may affect CSF pressure.

Although there are case reports that describe instances of acute syringomyelia, onset is generally chronic and slowly progressive. In some cases, syringomyelia is discovered incidentally on MRI in an asymptomatic patient; furthermore, patients can remain asymptomatic throughout their life and require no further follow-up or management. In the majority of cases, however, syringomyelia causes sufficient pressure on the spinal cord to cause a wide variety of symptoms. Most lesions develop in the cervical spinal cord and can expand superiorly into the brainstem, resulting in a syringobulbia, as well as inferiorly into the thoracic and even lumbar regions. As a result of the different syrinx types, spinal levels involved, degree of extension and diameter of lesion, symptomatology can be quite complicated and varied among patients.

Possible initial presenting symptoms include headache, severe segmental central and dysesthetic pain, loss of temperature and pain sensation, down-beating nystagmus, vocal cord dysmotility, urinary frequency and incontinence, stiffness, weakness, and scoliosis [5–7]. This varied presentation can lead to misdiagnosis.

Our patient initially developed unilateral upper extremity weakness that gradually progressed to involve the contralateral limb by the time he was first seen by a physician. For several years, despite the severity of weakness and muscular atrophy, his symptoms were confined to his upper extremities. Our patient received a diagnosis of muscular dystrophy despite developing a pattern of symptoms that did not correspond with this diagnosis. Over 20 years later, his examination and work-up demonstrated severe focal segmental wasting and weakness of his upper extremities in addition to mild weakness of his lower extremities, unilateral vocal cord paralysis, and paraspinal muscle atrophy. Findings on examination correlated with a central cord syndrome.

Given the topography of the weakness in his upper extremities with predominant involvement of his forearm and distal muscles, lower motor neuron disorders were considered. Hirayama disease, also known as monomelic amyotrophy, is a sporadic focal motor neuron disease that primarily affects young men in the second and third decades of life. Although it is characterized by its gradual onset of focal muscular atrophy like other motor neuron diseases, it differs in that symptoms typically plateau within a few years without further worsening of symptoms or development of new symptoms. As a result, Hirayama disease is often described as a benign motor neuron disease. The disease will commonly affect a single limb, usually an upper extremity. Nonetheless, unaffected limbs may also show evidence of reinnervation on nerve conduction studies (NCS) and EMG [8]. Similarly, bibrachial amyotrophy or flail arm syndrome is a variation of lower motor neuron disease that only affects the upper extremities. Compared to typical amyotrophic lateral sclerosis, flail arm syndrome also has a much better prognosis [9].

Once a syrinx is suspected, prompt imaging with MRI is important. Without early detection and treatment, some cases may progress such that spinal cord injury results in irreversible neurologic injury. Lesions are seen easily on brain and spine imaging, both in axial and sagittal planes, with several different magnetic resonance techniques [10]. NCS and EMG can also be used in the diagnosis of syringomyelia. CMAP amplitude diminishment, the presence of fibrillation potentials, chronic neurogenic motor unit potential (MUP) changes and reduced MUP recruitment in affected spinal segment levels can all provide clues to the diagnosis [11]. Our MRI findings of extensive syringohydromyelia from C1 to T11, NCS and EMG findings of severe motor neuronopathy in the cervical myotomes with sparing of sensory nerves, and his normal lower extremity NCS parameters and EMG findings did correlate with our patient’s clinical course and examination.

Treatment for syringomyelia is primarily surgical; however, not all cases require surgical intervention. Asymptomatic incidental lesions generally are followed clinically with periodic imaging, although there are centers where prophylactic surgery is performed [12]. Mild symptomatic cases of syringomyelia can remain stable and sometimes even gradually spontaneously resolve. Although controversial, mild cases are often treated conservatively as well [13]. In the case of symptomatic acquired syringomyelia, treatment of the underlying acquired cause is attempted before surgical intervention. Patients with clear symptoms are treated with surgical decompression to restore normal CSF flow. Several techniques exist, including posterior foramen magnum decompression with or without dural opening, anterior foramen magnum decompression, and shunting [7]. Patients usually stabilize or experience modest improvement in their symptoms following surgical correction of the underlying cause of the syrinx. Recurrence or residual syringomyelia following Chiari decompression in adults occurs in an average of 6.7% of cases. In cases of large holocord syringes, spinal cord injury may lead to permanent symptoms or disability, despite adequate decompression and reduction of the syrinx caliber [14].

Delay of diagnosis resulted in a severe gradual deterioration in our patient. His initial clinical diagnosis of muscular dystrophy was further confirmed with diagnostic studies, according to the family, although we acknowledge that another muscle biopsy may be needed to exclude a diagnosis of muscular dystrophy (though we doubt that this was the actual diagnosis). As a result of the original diagnosis, however, our patient did not seek further evaluation for several years because he understood that there was no treatment for his condition. Decades later, further work-up with simple imaging techniques easily confirmed the etiology of his symptoms. Unfortunately, this delay in diagnosis resulted in the development of irreversible severe chronic muscle wasting. With such advanced atrophy and severe weakness, surgery will likely not provide significant functional benefit.

The differential diagnosis of bibrachial atrophy and syringomyelia is important. While we cannot definitively exclude that both the cervicothoracic syringomyelia and the bibrachial amyotrophy occurred as two separate entities, we doubt this. We feel instead that our patient’s Chiari malformation type I with cervicothoracic syringomyelia masqueraded as bibrachial amyotrophy. This is supported by our electrodiagnostic findings, which did not show active denervation in both limbs but rather isolated active denervation in the right triceps brachii, along with the absence of muscle fasciculations and upper motor neuron signs on physical examination. Furthermore, the fact that our patient’s symptoms began relatively suddenly, at an earlier age then is typical for bibrachial atrophy, and his survival to date argue against him having bibrachial atrophy as a separate entity. Concerning the other important possibility of Hirayama disease, we also feel that this is unlikely as there was no neck pain and our patient eventually developed unilateral vocal cord paralysis, which is atypical in this disorder. Additionally, our patient progressed to develop contralateral limb paresis. This along with the aforementioned relatively sudden onset of his symptoms argue against monomelic amyotrophy (Hirayama disease).

We do not know why our patient’s symptoms were stable for over 20 years. Although there was no history of any preceding head or neck trauma, perhaps the syrinx rapidly enlarged in the process of the disease. Without prior imaging, this is impossible to say with any certainty.

Of interest, our patient did not complain of occipital or neck pain. This may be explained by findings in his recent sagittal MRI images. Despite showing the downward descent of the cerebellar tonsils, these images showed wide CSF spaces at the level of the foramen magnum, without signs of medullary compression. It would be helpful to show axial T2 images at the level of the foramen magnum to show the degree of brain stem compression, but unfortunately these were not available. We feel, however, that the other images suffice in this regard.

Much remains to be addressed in our understanding and management of syringomyelia. Despite multiple proposed theories for its pathogenesis, the development of intramedullary cysts within the spinal cord is still not completely understood. Recognition of key symptoms is often difficult given the nonspecific nature of presenting symptoms. Once diagnosed, a clear management strategy and surgical approach remains somewhat elusive. A greater awareness of this disorder is necessary among clinicians from a variety of disciplines given the potential for severe debilitation and difficult-to-treat pain syndromes in misdiagnosed or poorly managed cases.

## Conclusions

Syringomyelia is a potentially serious neurologic condition with vague and complicated symptoms that can mimic other neuromuscular disorders. Early detection and diagnosis with MRI is critical in preventing progression of spinal cord compression and potential neurologic injury. Symptomatic cases are generally treated surgically.

## Consent

Written informed consent was obtained from the patient for publication of this case report and any accompanying images. A copy of the written consent is available for review by the Editor-in-Chief of this journal.

## Authors’ information

JRM is a clinical neurophysiology fellow at the University of Southern California-Keck School of Medicine-Los Angeles County Medical Center. SRB is a professor of neurology at the University of Southern California-Keck School of Medicine-Los Angeles County Medical Center. SRB is Director of the University of Southern California Neuromuscular Program, a Fellow of the American Academy of Neurology and the American Association of Neuromuscular and Electrodiagnostic Medicine, and is board certified by the American Board of Psychiatry and Neurology in Neurology, Clinical Neurophysiology, Pain Medicine, and Neuromuscular Medicine. SRB is also board certified by the American Board of Electrodiagnostic Medicine in Electrodiagnostic Medicine. SRB is a member of the advisory board and the scientific committee of the Myasthenia Gravis Foundation of California. RAR is a Deputy Editor for the *Journal of Medical Case Reports*, an Associate Neurology Editor for *BMC Neurology*, *Grand Rounds* and *WebmedCentral*, and a Section Editor for *BMC Research Notes*. RAR practices general neurology at Neurology Consultants Medical Group, serves as Medical Director of the PIH Health Stroke Program, is a Clinical Assistant Professor of Neurology at the University of Southern California-Keck School of Medicine-Los Angeles County Medical Center, and is a Fellow of the American Association of Neuromuscular and Electrodiagnostic Medicine. RAR is board certified by the American Board of Psychiatry and Neurology in Neurology and Vascular Neurology, and Neurocritical care and Neuroimaging by the United Council of Neurologic Subspecialties. RAR is also board certified by the American Board of Electrodiagnostic Medicine in Electrodiagnostic Medicine. RAR is a former president of the Los Angeles Neurological Society, and is a Fellow of the American Academy of Neurology and a Fellow of the American Neurological Association.

## Abbreviations

CMAP: compound muscle action potentials
CSF: cerebrospinal fluid
EMG: electromyography
MRI: magnetic resonance imaging
NCS: nerve conduction studies
MUP: motor unit potential
SNAP: sensory nerve action potentials.

## References

[1] Milhorat TH: **Classification of syringomyelia.** *Neurosurg Focus* 2000,**8**(3)**:**E1. 10.3171/foc.2000.8.3.1 16676921

[2] Stoodley MA, Jones NR, Yang L, Brown CJ: **Mechanisms underlying the formation and enlargement of noncommunicating syringomyelia: experimental studies.** *Neurosurg Focus* 2000,**8**(3)**:**E2. 10.3171/foc.2000.8.3.2 16676925

[3] Brickell KL, Anderson NE, Charleston AJ, Hope JK, Bok AP, Barber PA: **Ethnic differences in syringomyelia in New Zealand.** *J Neurol Neurosurg Psychiatry* 2006,**77**(8)**:**989–991. 10.1136/jnnp.2005.081240 10.1136/jnnp.2005.081240 PMC2077633 16549414

[4] Koyangi I, Houkin K: **Pathogenesis of syringomyelia associated with Chiari type 1 malformation: review of evidences and proposal of a new hypothesis.** *Neurosurg Rev* 2000, **33:**271–285. 10.1007/s10143-010-0266-5 20532585

[5] Todor DR, Mu HTM, Milhorat TH: **Pain and syringomyelia: a review.** *Neurosurg Focus* 2000,**8**(3)**:**E11. 10.3171/foc.2000.8.3.11 16676923

[6] Paul KS, Lye RH, Strang FA, Dutton J: **Arnold-Chiari malformation. Review of 71 cases.** *J Neurosurg* 1983,**58**(2)**:**183–187. 10.3171/jns.1983.58.2.0183 10.3171/jns.1983.58.2.0183 6848674

[7] Alden TD, Ojemann JG, Park TS: **Surgical treatment of Chiari I malformation: indications and approaches.** *Neurosurg Focus* 2001,**11**(1)**:**E2. 10.3171/foc.2001.11.1.3 16724812

[8] Verms S, Bradley WG: **Atypical motor neuron disease and related motor syndromes.** *Semin Neurol* 2001,**21**(2)**:**177–188. 10.1055/s-2001-15263 10.1055/s-2001-15263 11442326

[9] Couratier P, Truong CT, Khalil M, Deviere F, Vallat JM: **Clinical features of flail arm syndrome.** *Muscle Nerve* 2000, **23:**646–648. 10.1002/(SICI)1097-4598(200004)23:4<646::AID-MUS26>3.0.CO;2-E 10.1002/(sici)1097-4598(200004)23:4<646::aid-mus26>3.0.co;2-e 10716778

[10] Roser F, Ebner FH, Sixt C, Hagen JM, Tatagiba MS: **Defining the line between hydromyelia and syringomyelia. A differentiation is possible based on electrophysiological and magnetic resonance imaging studies.** *Acta Neurochir* 2010,**152**(2)**:**213–219. 10.1007/s00701-009-0427-x 10.1007/s00701-009-0427-x 19533016

[11] Veilleux M, Stevens JC: **Syringomyelia: electrophysiologic aspects.** *Muscle Nerve* 1987,**10**(5)**:**449–458. 10.1002/mus.880100512 10.1002/mus.880100512 3614259

[12] Navarro R, Olavarria G, Seshadri R, Gonzales-Portillo G, McLone DG, Tomita T: **Surgical results of posterior fossa decompression for patients with Chiari I malformation.** *Childs Nerv Syst* 2004,**20**(5)**:**349–356. 10.1007/s00381-003-0883-1 10.1007/s00381-003-0883-1 15022006

[13] Novegno F, Caldarelli M, Massa A, Chieffo D, Massimi L, Pettorini B, Tamburrini G, Di Rocco C: **The natural history of the Chiari Type I anomaly.** *J Neurosurg Pediatr* 2009,**2**(3)**:**179–187. 10.3171/PED/2008/2/9/179 18759599

[14] Schuster JM, Zhang F, Norvell DC, Hermsmeyer JT: **Persistent/recurrent syringomyelia after Chiari decompression – natural history and management strategies: a systematic review.** *Evid Based Spine Care J* 2013,**4**(2)**:**116–125. 10.1055/s-0033-1357362 10.1055/s-0033-1357362 PMC3836893 24436709

